# Thalamic shape and volume abnormalities in female patients with panic disorder

**DOI:** 10.1371/journal.pone.0208152

**Published:** 2018-12-19

**Authors:** Takeshi Asami, Haruhisa Yoshida, Masao Takaishi, Ryota Nakamura, Asuka Yoshimi, Thomas J. Whitford, Yoshio Hirayasu

**Affiliations:** 1 Department of Psychiatry, Graduate School of Medicine, Yokohama City University, Yokohama, Japan; 2 School of Psychology, University of New South Wales, Sydney, New South Wales, Australia; 3 Heian Hospital, Urazoe, Japan; Chiba Daigaku, JAPAN

## Abstract

The thalamus is believed to play crucial role in processing viscero-sensory information, and regulating the activity of amygdala in patients with panic disorder (PD). Previous functional neuroimaging studies have detected abnormal activation in the thalamus in patients with PD compared with healthy control subjects (HC). Very few studies, however, have investigated for volumetric abnormalities in the thalamus in patients with PD. Furthermore, to the best of our knowledge, no previous study has investigated for shape abnormalities in the thalamus in patients with PD. Twenty-five patients with PD and 25 HC participants (all female) were recruited for the study. A voxel-wise volume comparison analysis and a vertex-wise shape analysis were conducted to evaluate structural abnormalities in the PD patients compared to HC. The patients with PD demonstrated significant gray matter volume reductions in the thalamus bilaterally, relative to the HC. The shape analysis detected significant inward deformation in some thalamic regions in the PD patients, including the anterior nucleus, mediodorsal nucleus, and pulvinar nucleus. PD patients showed shape deformations in key thalamic regions that are believed to play a role in regulating emotional and cognitive functions.

## Introduction

Panic disorder (PD) is characterized by recurrent unexpected panic attacks. Symptoms of panic attacks include palpitations, feelings of choking, chest pain, and fear of dying. PD is related with high levels of social, occupational, and physical disability, and considerable economic cost [1, 2]. PD has been also thought to be associated with increased suicide risk [3]. In light of its substantial social, occupational, and physical burden, it is necessary to clarify neuroanatomical basis of PD.

Recent neuroimaging studies have detected structural and functional abnormalities in some brain regions, such as limbic and prefrontal regions, in patients with PD compared with healthy control subjects (HC). Specifically, with regards to previous structural neuroimaging studies, patients with PD exhibit smaller gray matter volume in amygdala, which is a principal region involved in anxiety, and the medial prefrontal cortex (including the cingulate gyrus), which is believed to be involved in regulating the activity of the amygdala [4–7]. In addition, other cerebral regions, such as insula, superior temporal gyrus, and orbitofrontal gyrus, have also been demonstrated to have smaller volumes in the patients with PD compared with HC [8–11]. These regions are believed to have emotional and cognitive functions, and to play a role in the regulation of the amygdala. On the other hand, patients with PD have been reported to have abnormally large volumes in the brain stem regions, which receives efferent projections from the amygdala [12, 13]. Thus, several brain regions, especially those involved in emotional and cognitive function, are believed to play a role in the development of PD. The present study will focus on the relationship between structural abnormalities in the thalamus and the development of PD. Our interest in the thalamus stems from the fact that the thalamus has anatomical and functional connections with all of the brain regions implicated above. Consequently, the thalamus has been hypothesized to be a key regions for neurobiological basis of PD [14]. In addition, previous studies have reported structural abnormalities in the thalamus in patients with other anxiety disorders, such as generalized anxiety disorder [15] and social anxiety disorder [16].

The thalamus consists of several nuclei, including the anterior nucleus, mediodorsal nucleus, and pulvinar nucleus. Each nucleus has connections with various other brain regions and, therefore, the thalamus is believed to be involved in various functions, including emotion, cognition, and the control of motor actions [17]. The thalamus has reciprocal connections with the amygdala and medial prefrontal cortex, and is thus believed to be involved in the production and regulation of anxiety and fear. According to a neuroanatomical hypothesis of PD [14], the thalamus is thought to play a role in both downstream and upstream pathways that are used for conveying viscerosensory information from the nucleus of the solitary tract to the amygdala. In the downstream pathway, viscerosensory information is transferred from the nucleus of the solitary tract to the amygdala via the sensory thalamus, while in the upstream pathway, the information is conveyed through the corticothalamic pathway including thalamus, insula, cingulate gyrus, and medial prefrontal cortex [14]. In summary, it has been suggested that the thalamus has crucial role in the neurobiological basis of PD, via the following mechanism: a failure in processing viscerosensory information in the thalamus may cause abnormal amygdala activation, resulting in appearance of PD symptoms. In support of this account, several recent neuroimaging studies have demonstrated functional abnormalities in thalamus in patients with PD compared with HC [18–22]. However, there has, to the best of our knowledge, been only one study which has found structural abnormalities in the thalamus in patients with PD, which is from our laboratory [8]. According to the study, significant gray matter volume reductions were observed in the thalamus only in female patients with PD (i.e., not in male patients with PD), compared with the matched HC.

There are some previous studies which have reported sex differences in epidemiological data and in regional brain activities for emotional stimuli in PD. Epidemiological studies have described that the lifetime prevalence of PD is more than twice as high in women compared to men [23]. Furthermore, recurrence of panic symptoms after remission are higher among female compared to male patients with PD [24]. In addition, a previous report has revealed that while the highest rate for panic attacks among men is in the age range of 15–24 years, for women the peak range is from 35–44 years [25]. An experimental study has demonstrated that women reported more fear and panic relative to men after panicogenic inhalation of 20% CO_2_ enriched air [23]. In terms of previous neuroimaging studies, a functional magnetic resonance image (MRI) study reported significantly stronger activations in various brain regions, including the thalamus, in the female patients with PD compared with male patients during facial emotion processing [26]. Another study also identified functional abnormalities in female participants at risk for PD: the PD patients showed higher activity in the thalamus during interoception, relative to the HC participants [27].

Based on these previous studies which identified sex differences in PD, in the current study, we evaluated structural changes in the thalamus in female patients with PD (only), compared with matched HC. A shape analysis was conducted in addition to a regular volume comparison analysis using voxel-based morphometry (VBM). In the shape analysis, subcortical brain regions (e.g., thalamus) are segmented based on their shapes and intensity variations. Shape analysis measures changes of the morphology directly, and, unlike the VBM, smoothing procedure is not demanded. It has been, thus, thought that regional alterations of the subcortical regions are detected more precisely in the shape analysis relative to the VBM [28, 29]. Indeed, previous shape analyses have demonstrated localized structural abnormalities, which were not detected in the VBM analysis, in some subcortical regions in neurological and psychiatric disorders [29–32].

In our analysis, we hypothesized that changes in thalamus shape would be confirmed in the specific nuclei that are believed to be involved in regulating emotional and cognitive function, including the anterior, mediodorsal, and pulvinar nuclei [17], in the female patients with PD.

## Materials and methods

### Subjects

Twenty-five female patients with PD and 25 female HC were included in this study. Female patients with PD were recruited from Yokohama City University Hospital, and HC were recruited from the community and hospital staff. All the participants had participated in our previous study [33], and of those participants, fifteen female patients and fifteen female HC had also taken part in our previous VBM study [8]. Exclusion criteria for the study were non-right-handedness, a history of seizures, head trauma with loss of consciousness, neurological disorders and/or a history of substance abuse. Patient diagnosis was confirmed based on the Structured Clinical Interview for DSM-IV Axis I Disorders (SCID-I) [34]. HC were confirmed to have no Axis I disorders using the SCID Non-patient Edition [34] and the Mini-International Neuropsychiatric Interview [35]. Participants’ socio-economic status (SES) and their parental SES were assessed using the Hollingshead Two-Factor Index [36]. Patients’ illness severity and general functioning were evaluated using the Panic Disorder Severity Scale (PDSS) [37] and the Global Assessment of Functioning (GAF). Six patients had a past history of major depression often related to PD [2], but had no current or past history of other psychiatric disorders. Patients had been receiving selective serotonin reuptake inhibitors (SSRIs) alone (n = 3); benzodiazepines alone (n = 3); SSRIs and benzodiazepines (n = 15); serotonin-norepinephrine reuptake inhibitors and benzodiazepines (n = 1); and tricyclic antidepressants and benzodiazepines (n = 1). This study was approved by the Medical Research Ethics Committee of Yokohama City University. After providing a complete description of the study, we obtained written informed consent from all participants.

### MRI processing

MRI data were obtained using a 1.5-T Magnetom Symphony system (Siemens Medical System, Erlangen, Germany) at Yokohama City University Hospital. A series of 128 contiguous T1-weighted images in sagittal section was acquired with a Turbo FLASH sequence. The parameters for MR imaging were: echo time = 3.93ms, repetition time = 1960ms, inversion time = 1100ms, field of view = 24cm, flip angle = 15, matrix = 256x256x128, and voxel dimensions = 0.9375x0.9375x1.5mm.

### Voxel-based morphometry: Small volume correction analysis

The theory and algorithms underlying VBM (as implemented with the Statistical Parametric Mapping (SPM)12 software; Wellcome Department of Cognitive Neurology, London, UK) have been well-documented [38]. VBM was conducted using the Diffeomorphic Anatomical Registration Through Exponentiated Lie algebra (DARTEL) tool in SPM12 [39]. The detailed protocol for our VBM processing pipeline has been described in our previous report [40]. Briefly, T1-weighted images were first segmented into probability maps of gray and white matter, and then, a population gray matter template was created using DARTEL. Second, the gray matter probability maps of each subject were non-linearly normalized to the population template, and Jacobian modulated. Third, the population gray matter template was affine transformed into MNI space, and all the individual gray matter maps in the template space were then co-registered to MNI space using the same transformation. Finally, smoothing was conducted with a Gaussian kernel of 8-mm full-width at half-maximum.

For evaluating group differences in thalamic volume, a general linear model was employed. A two-sample t-test with small-volume correction (SVC) for bilateral thalamus was conducted using WFU Pickatlas software [6, 41]. Age and intracranial content (ICC) volumes, calculated using a MATLAB function, were entered as confounding covariates [40]. Significance level was set at p < .05 (corrected for family-wise error). Once significant differences were confirmed, regional volumes were calculated as per the procedure of [40, 42]. Correlation analyses were conducted between these volumes and patients’ scores on the PDSS and GAF. A critical p-value of .05 was used for this analysis.

### Shape analysis

Shape analysis for thalamus were conducted using FMRIB’s Integrated Registration Segmentation Toolkit (FIRST) in FSL (version 5.0.10) [28]. FIRST is a model-based automated registration/segmentation tool. First, the thalamus was extracted bilaterally from the T1-weighted images using FIRST. The deformable surfaces of the thalamus were used to automatically parameterize the volumetric labels for meshes. This surface mesh was composed of a fixed set of connected vertices that corresponded to each participant. This made it possible to compare the shape of the thalamus between groups. The normalized intensities along the mesh surface were then sampled and modeled. Finally, the surface meshes used in the statistical analysis were reconstructed in MNI space.

A general linear model approach was applied to assess group differences in the shape of thalamus. A permutation-based inference tool for nonparametric statistics was used, and a two-sample t-test with ICC volume and age as covariates was conducted for vertex-wise group comparisons using a threshold-free cluster enhancement method [43, 44]. The number of permutations was set at 5000, and the significance level was set at p < .05 (corrected for False-Discovery Rate) [29]. In the case that significant group-wise differences were found, a mean vertex-level scalar projection value (which represent the degree of inversion or eversion from the average thalamus shape) was calculated as per the procedure of [45]. Correlation analyses were conducted between these values and patients’ scores of the PDSS and GAF. A critical p-value of .05 was used for this analysis.

## Results

There were no significant group differences in participants’ age, SES, or parental SES (**Table 1**).

**Table 1:** Demographic and clinical characteristics of the study groups.

	PD group		HC group				
	(n = 25)		(n = 25)				
Variable	Mean	SD	Mean	SD	df	t	p
Age (years)	39.7	9.9	39.0	11.1	48	0.24	.81
[range]	[23–56]		[22–58]				
Participant’s SES ^a^	2.7	0.9	2.5	0.8	48	0.66	.52
Parental SES ^a^	2.8	0.8	2.4	0.9	48	0.96	.20
PDSS Score	12.3	5.5			25		
GAF Score	61.0	9.3			25		
Duration of illness (years)	4.7	5.8			25		

Abbreviations: PD, panic disorder; HC, healthy control subject; SES, socioeconomic status; PDSS, Panic Disorder Severity Scale; GAF, Global Assessment of Functioning

^a^ Higher scores mean lower socioeconomic status

### Voxel-based morphometry: Small volume correction analysis

**Fig 1:**
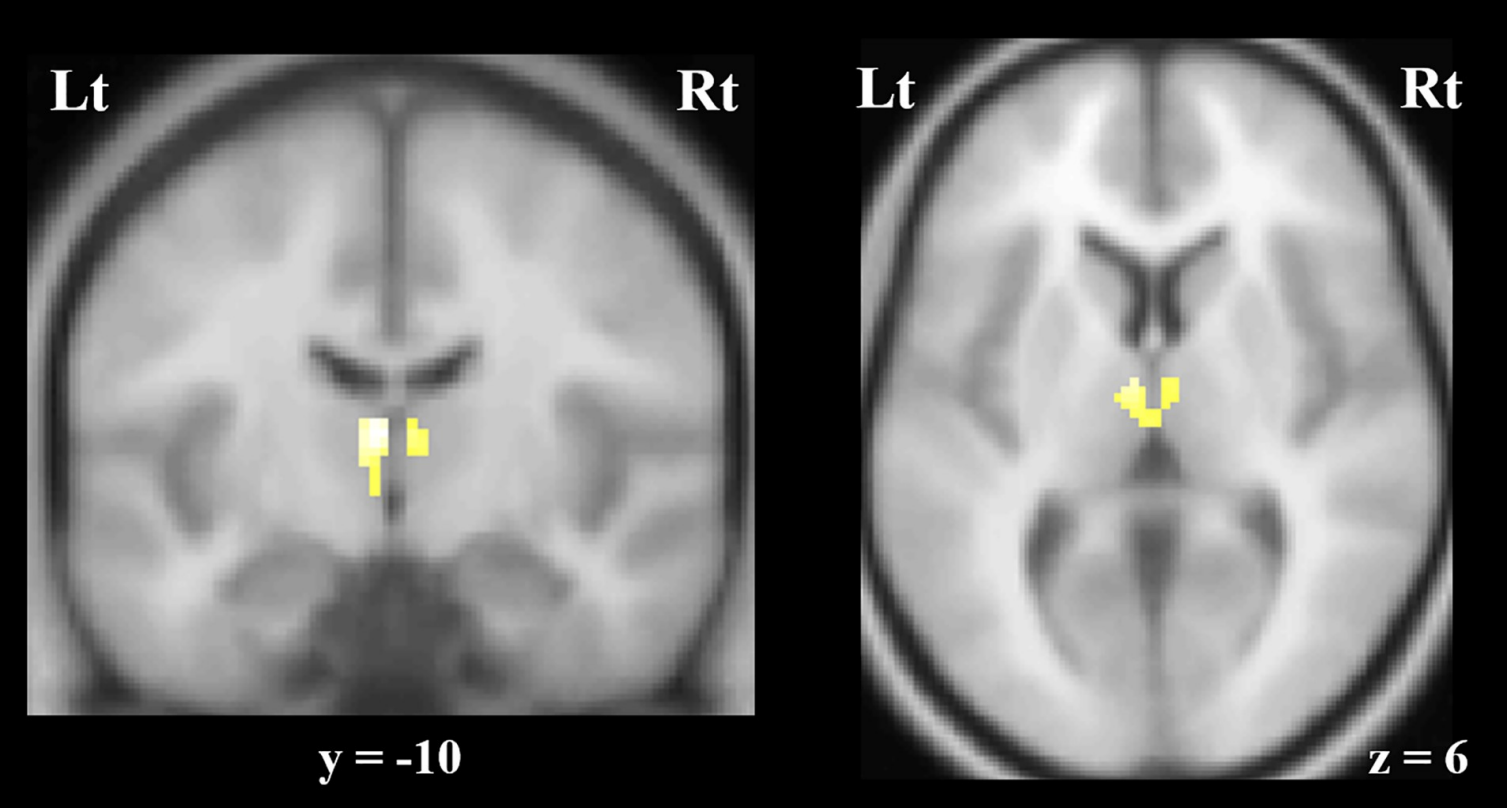
The voxel-based morphometry (VBM) analysis showed significant gray matter volume reductions in the anterior medial part of the thalamus, bilaterally, in the female patients with panic disorder compared to female healthy control participants (family-wise error corrected, P < .05). Abbreviations: Rt, right; Lt, left.

The VBM-SVC analysis revealed that the female patients with PD had significant gray matter volume reductions in the thalamus bilaterally, compared with the female HC. Specifically, these regions of reduction were observed in the anterior medial part of the thalamus, bilaterally (Peak coordinate: (x, y, z = -4, -10, 10), Family-wise error corrected P = .004 (cluster-level) and .002 (peal-level), voxel size = 142) (**Fig 1**). Correlation analyses showed no significant associations between these volumes and scores on the PDSS or GAF in the patients with PD.

### Shape analysis

**Fig 2:**
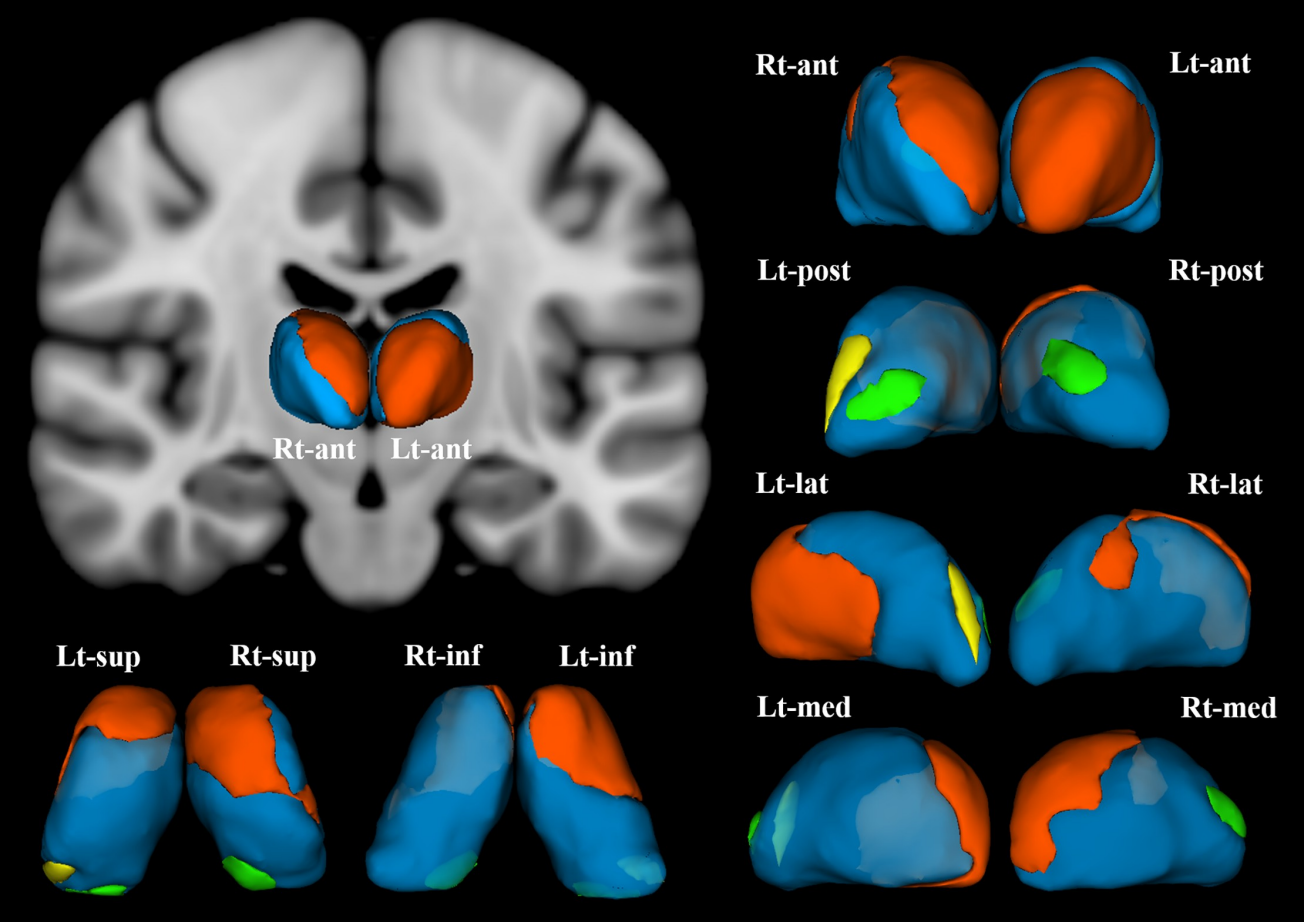
Female patients with panic disorder showed significant inward deformation of shape in the bilateral thalamus compared with female healthy control subjects (false discovery rate corrected, P < .05). These regions included (in the right thalamus) the anterior nucleus, medial mediodorsal nucleus, and lateral posterior nuclei (orange), and the medial part of pulvinar nucleus (green). In the left thalamus, the anterior nucleus, ventro-lateral nucleus, ventral anterior nucleus (orange), the medial part of pulvinar nucleus (green), and the lateral part of pulvinar nucleus (yellow) were affected. Abbreviations: Rt, right; Lt, left; ant, anterior view; post, posterior view; lat, lateral view; med, medial view; sup, superior view; inf, inferior view; 3D-images were created using 3D-Slicer.

The shape analysis revealed that the female patients with PD demonstrated significant inward deformation of shape in the thalamus bilaterally compared with the female HC. Specifically, these regions of the inward deformation included the anterior nucleus, mediodorsal nucleus, lateral posterior nucleus, and medial part of pulvinar nucleus of the right thalamus, and the anterior nucleus, ventro-lateral nucleus, ventral anterior nucleus, and the medial and lateral parts of pulvinar nuclei of the left thalamus (**Fig 2**). The degree of deformation in each thalamic region was not significantly correlated with scores on the PDSS or GAF in the patients with PD.

## Discussion

The shape analysis component of the present study provided new evidence that female patients with PD show significant inward deformation of shape in certain regions in the thalamus. To the best of our knowledge, this is the first study reporting structural abnormalities in the shape of thalamus in patients with PD. Furthermore, the volumetric analysis of the present study also revealed significant gray matter volume reductions in the thalamus bilaterally in the female patients with PD, compared with the matched female HC.

In the current study, we first confirmed that the female patients with PD had significant gray matter volume reductions in the bilateral thalamus, relative to the female HC. In our previous VBM analysis which reported sex differences in regional gray matter volumes in patients with PD, gray matter volume reductions in thalamus were identified only in the female patients but not in the male patients with PD [8]. With regards to previous studies from other laboratories: as far as we know, there have been no previous region-of-interest studies which have investigated for volumetric abnormalities in the thalamus in patients with PD. While some previous studies have investigated for volumetric abnormalities across the whole brain in patients with PD (using VBM), none have reported volumetric changes in the thalamus [4, 7, 9, 10, 13, 46, 47]. One possible reason for why these studies failed to observe volumetric changes in the thalamus was a lack of statistical power; the majority of these studies had group sizes of less than 20 participants [4, 7, 10, 13, 46]. A second possibility was that some studies included both female and male patients with PD, and thus any sex-specific effects on regional brain volume changes might be diminished [9, 47]. In light of previous evidence of sex differences in PD (both in terms of epidemiology and symptomatology [23, 24]), a more fruitful approach might be to investigate structural brain changes in female and male patents with PD in separate studies.

In contrast to relatively limited evidence for thalamic abnormalities in structural neuroimaging studies, there have been numerous previous studies which have reported functional abnormalities in the thalamus in patients with PD. For example, a single-photon emission computed tomography study reported decreased serotonin transporter binding in the thalamus in patients with PD [19]. A positron emission tomography study also observed PD patients to show higher levels of glucose uptake in the bilateral thalamus relative to HC [18]. Some task-related functional MRI studies have also demonstrated abnormal BOLD activity in the thalamus in patients with PD compared with HC [20–22]. Moreover, recent resting state functional MRI studies have reported functional abnormalities in the thalamus in PD patients, even while patients were in remission following pharmaceutical intervention with escitalopram [48, 49]. Finally, there is also a functional MRI study which showed sex differences in the functional connectivity between amygdala and thalamus during the processing of angry facial expressions in PD patients. Specifically, this study observed greater functional connectivity in the female PD patients compared to the male PD patients [26].

The results of our volumetric analysis, combined with the results of the previous functional imaging studies described above, suggest that structural abnormalities in the thalamus may play an important role in the etiology of PD. However, while our VBM-SVC analysis demonstrated significant gray matter volume reductions in the anterior medial part of thalamus in the female patients with PD, there is need to evaluate structural changes in thalamus more minutely. This is because the thalamus consists of several discrete nuclei, each having different anatomical connections and functions. For the purpose of detecting minute structural change, vertex-wise shape analysis has been believed to have advantages relative to voxel-wise volumetric analysis [28, 29]. To this end, the current shape analysis demonstrated significant inward deformation of shape in specific thalamic nuclei, in the female patients with PD compared to the female HC. These regions included the anterior nucleus, mediodorsal nucleus, lateral posterior nucleus, and the medial part of pulvinar nucleus of the right thalamus, and the anterior nucleus, ventro-lateral nucleus, ventral anterior nucleus, and the medial and lateral parts of pulvinar nuclei of the left thalamus.

The anterior nuclei of the thalamus receive neuronal input from the limbic system, including the hippocampus and mammillary bodies. These nuclei also have connections with anterior cingulate gyrus [17]. Consistent with their structural connections, the anterior nuclei are believed to play a functional role in memory and affective cognition [50]. In regards to the neural basis of PD, the anterior cingulate gyrus has been thought to have crucial role for regulating amygdala activity. Thus, it is possible that structural deficits in the anterior nucleus of the thalamus might cause functional abnormalities in the cingulate gyrus, resulting in excessive activation of amygdala in PD patients.

The mediodorsal nuclei of the thalamus are believed to have structural connections with the amygdala, which is a principal region for the neurobiology of anxiety disorders including PD. It is also structurally connected with the prefrontal cortex, which plays a key role in controlling the activity of the amygdala. The mediodorsal nuclei are believed to play a functional role in emotion, cognition, and learning [17]. It has been thought that activity of amygdala is regulated by prefrontal cortex directory or via thalamus [51]. Thus, it is possible that structural abnormalities in the mediodorsal nucleus of the thalamus might lead to a misregulation of prefrontal cortex activity, with a corresponding downstream effect on amygdala activity.

The lateral posterior nuclei have structural connections with the frontal and parietal cortices. They are believed to be functionally involved in the interpretation and integration of sensory information and emotions. Thus, it is feasible that structural abnormalities in these nuclei could lead to emotional dysfunction. The ventral anterior nucleus and ventro-lateral nucleus are well-known to be involved in motor function, however recent reports have also described their possible association with anxiety. For example, a recent resting stare functional MRI study revealed that the fractional amplitude of low-frequency fluctuations–which are an indicator for the intensity of regional brain spontaneous activities–were decreased in the right ventral lateral nucleus of thalamus in patients with PD compared with HC [52]. A recent case report also described that levels of anxiety were decreased by deep brain stimulation of the ventral anterior and anterior nuclei in patients with obsessive compulsive disorder [53].

The medial portion of the pulvinar nucleus has reciprocal connections with multimodal sensory association areas, including the amygdala, insula, cingulate cortex, orbitofrontal cortex and superior temporal gyrus [54]. All of these regions have been observed to show gray matter volume reductions in patients with PD compared with HC [5, 6, 8–11, 13]. It has been thought that the medial part of pulvinar nucleus is related to fear-related cognitions, and early activation of amygdala in response to masked fear-provoking stimuli is mediated via the pulvinar-amygdala pathway. It is thus possible that structural deficits in medial parts of pulvinar nucleus could cause abnormalities in fear-related cognitions which could, in turn, lead to abnormal activity in amygdala in patients with PD.

In summary, the current study demonstrated structural abnormalities in thalamus, especially in the nuclei associated with anxiety, in the female patients with PD compared with female HC. There are, however, some limitations worth noting in this study. Firstly, our sample size, while larger than the majority of previous MRI studies of PD, was still relatively small (n = 25 per group). Secondly, almost all the female patients were receiving psychoactive medication, which could have influenced the results. Future studies with larger sample sizes and using drug naïve patients would be worthwhile. Third, the present study only investigated female patients with PD. While this was an advantage in that it increased the homogeneity of the participant sample, future studies should also evaluate structural abnormalities in thalamus should in male patients, in order to better understand the basis of sex differences in the neuroanatomical underpinnings of PD. Finally, recent neuroimaging studies have segmented the thalamus on the basis of information from thalamo-cortical tractography [55, 56]. It would be worthwhile confirming our results using both diffusion tensor images and higher resolution 3T-MRI data in future research.

In conclusion, the VBM-SVC analysis of the present study demonstrated gray matter volume reductions in the thalamus bilaterally in the female patients with PD compared with matched female HC. Furthermore, the shape analysis revealed that the female patients showed significant inward deformation of shape in certain thalamic regions, including the anterior, mediodorsal, and pulvinar nuclei of the thalamus. These results suggest that structural abnormalities in thalamus may play an etiological role in the development and maintenance of PD.
